# Rutin Concentration and Characterization of Rutinosidase in Perennial Buckwheat (*Fagopyrum cymosum*) and Its Application in Foods

**DOI:** 10.3390/foods12071417

**Published:** 2023-03-27

**Authors:** Tatsuro Suzuki, Rio Kurokoh, Shogo Murakami, Naohiro Takahashi, Asana Matsuura, Kenjiro Katsu, Kouhei Murata

**Affiliations:** 1 National Agriculture and Food Research Organization Kyushu Okinawa Agricultural Research Center, Suya 2421, Koshi, Kumamoto 861-1192, Japan; kkatsu9699@naro.affrc.go.jp; 2 School of Agriculture, Tokai University, 9-1-1 Toroku, Higashi-ku, Kumamoto-shi, Kumamoto 862-8652, Japankmurata@agri.u-tokai.ac.jp (K.M.); 3 Faculty of Agriculture, Shinshu University, 8304, Minamiminowa-Village, Kamiina-County Nagano, Nagano 399-4598, Japan; asana@shinshu-u.ac.jp

**Keywords:** rutinosidase, rutin, rutin hydrolysis, quality, bitterness, perennial buckwheat

## Abstract

To evaluate the potential of perennial buckwheat (*Fagopyrum cymosum*; FC) as a food source, rutin concentration was investigated. FC contains more than 1% (*w*/*w*) rutin and 0.03% quercetin in the leaves, flowers, and seeds. In particular, rutin and quercetin concentrations were high in plant seeds. Therefore, FC is useful as a rutin- and quercetin-rich material. In contrast, the FC seed contained a large amount of rutinosidase. Purified rutinosidase in a homogenous mixture consisted of only one isozyme with M.W. of 58.4 KD and low *K*_m_ for rutin (0.367 mM). The rutin concentration in the FC dough decreased to almost zero, 10 min after the addition of water. Parallel to the decrease in rutin, quercetin was increased, and strong bitterness was generated, whereas steam-heated flour in which rutinosidase was inactivated did not have rutin hydrolysis and bitterness. These results indicate that rutinosidase is a major cause of rutin hydrolysis and bitterness. The in vitro rutinosidase is inactivated at pH 8.0 and 65 °C. Therefore, the control of dough pH and temperature should be useful in preventing rutinosidase activity.

## 1. Introduction

Rutin is a flavonoid that has several beneficial effects, such as strengthening fragile blood capillaries [1] and antioxidation [2]. Buckwheat is the only known cereal that contains rutin. In addition, buckwheat contains a large amount of rutin not only in its seeds but also in its leaves [3]. For these reasons, buckwheat has been utilized as a material for rutin-rich food products [4]. Among buckwheat species, some studies have demonstrated that perennial buckwheat (*Fagopyrum cymosum* Meisn., FC) (Figure S1A) also contains rutin in its leaves [5]. Perennial crops have recently attracted attention because they have promising traits for sustainable food production, such as resistance to water/drought stress, pest regulation, soil carbon sequestration, and soil erosion [6]. Among perennial crops, perennial wheat “Kernza” (*Thinopyrum intermedium*) is well known [7]. However, perennial rice is also planted in tropical regions [8]. These perennial wheat and rice varieties have limited cultivation areas; they cannot grow in temperate zones that have a lot of rain (over 1000 mm rainfall per year) and soil freezing.

On the other hand, FC has mainly grown naturally in the western part of Japan, suggesting that it is strongly resistant to water stress and soil freezing; therefore, it can grow in the above-mentioned temperate zone. However, there are only a few reports that have investigated FC in the use of functional compounds such as rutin in terms of organ distribution. In addition, our preliminary study showed that FC has a critical demerit for food usage; its flour and processed food have strong bitterness. Strong bitterness is an important unfavorable characteristic that is widely rejected by both people and animals [9]. Therefore, the high bitterness of Tatary buckwheat seeds has prevented their widespread use. Tartary buckwheat (*F. tartaricum* Gaertn., FT) is also known to have strong bitterness in its flour and processed foods; therefore, it is referred to as “bitter buckwheat”. In addition, rutin in FT flour is hydrolyzed immediately after the addition of water [10]. Several studies have demonstrated that rutinosidases catalyze rutin hydrolysis in FT [10,11]. FT rutinosidase has markedly high activity, which is sufficient to hydrolyze the rutin present in buckwheat flour (approximately 1–2% (*w*/*w*)) to quercetin and rutinosidase. In addition, rutin hydrolysis is a major cause of strong bitter taste [12,13]. Quercetin is considered as one of the bitter compounds in Tartary buckwheat [11]. Therefore, rutinosidase is an unfavorable trait for food production. To date, there have been no reports demonstrating the cause of bitterness in FC. In addition, there are no reports showing whether rutin in FC is hydrolyzed after the addition of water to flour. This information is important for evaluating the potential of FC foods.

In this study, we evaluated rutinosidase activity in FC, followed by purification and enzymatic characterization. We demonstrated that rutinosidase in FC seeds causes rutin hydrolysis and bitterness generation after the addition of water to flour. In addition, we investigated the organ distribution of rutin and rutinosidase in FC, such as seeds, flowers, leaves, and roots, through which FC can be used as rutin-rich foods using appropriate plant organs. We also compared the above organ distributions with those of common buckwheat (*F. esculentum* Moench; FE) and FT to demonstrate that FC has sufficient potential as a rutin-rich material. These results provide useful information for the application of FC in sustainable food production.

## 2. Materials and Methods

### 2.1. Plant Materials

We show a flowchart of the experiments in Figure S1B. To investigate organ distribution of buckwheat species, the following varieties/germplasms were employed; “HITACHIAKISOBA” (FE), “Hokkai T8” (FT), “COL/NEPAL/1985/3079/OPEN” (FC). HITACHIAKISOBA is one of the most famous varieties of FE in Japan, with an intermediate autumn ecotype. The “Hokkai T8” is famous FT in Japan with summer ecotype and strong rutinosidase activity. The “COL/NEPAL/1985/3079/OPEN” is a germplasm introduced by FC from Nepal. These buckwheat cultivars were sown on 6th April 2020, in the experimental field of the National Agriculture and Food Research Organization Kyushu Okinawa Agricultural Research Center, Japan (32°52′37.88″ N/130°44′14.98″ E/altitude 337 m) for three replicates. Leaves and stems were harvested during the vegetative growth period, 21 d after germination. Flowers and roots were harvested at flowering time, approximately 35 d after germination. Seeds were harvested at maturation, when approximately 80% seeds turned from green to brown. The harvested samples were lyophilized (FDU-1200, EYELA, Tokyo, Japan) immediately after harvesting and milled into fine powder using a motor (AS ONE, Tokyo, Japan). For purification of rutinosidase from FC seeds, seeds were harvested at harvesting time, dried at 25 °C for 1 week, and then dehulled by hand and milled into fine powder using a motor.

### 2.2. Organ Distribution of Rutin, Quercetin, and Rutinosidase Activity in FC

To investigate rutin, quercetin concentration, and rutinosidase activity, the harvested organs described in Section 2.1 were used. Rutin concentration and rutinosidase activity were measured using the method of Suzuki et al. [14], with minor modifications. Briefly, 30 mg of flour was mixed with 80% (*v*/*v*) methanol containing 0.1% (*v*/*v*) phosphoric acid and extracted for 24 h at 60 °C. The centrifuged (MK-301, TOMY, Tokyo, Japan) supernatant was passed through a 0.45 μm PTFE hydrophobic filter (Advantec, Tokyo, Japan) and then applied to an ultra-high performance liquid chromatography (UPLC) system (Waters, Tokyo, Japan) to separate rutin and quercetin. To investigate in vitro rutinosidase activity, a standard assay was performed by measuring the quercetin concentration in the reaction mixture using UPLC. The standard assay mixture consisted of 50 mM acetate-LiOH (pH 5.0, 4 °C), 20% (*v*/*v*) methanol and 0.2% (*w*/*v*) rutin in a final volume of 0.05 mL. The reaction was performed at 37 °C and was stopped by adding 0.2 mL methanol, and then, we investigated quercetin concentration, which is the product of rutinosidase activity, using UPLC [15]. The above measurements were performed in three independent replicates. To investigate the tissue distribution of FC rutinosidase in seeds, testa, endosperm, and embryo were carefully separated using tweezers according to a previous study on FT seeds [11]. Separated tissue was homogenized to flour using a motor and used for the analysis of rutin concentration and rutinosidase activity.

### 2.3. Purification and Characterization of Rutinosidase from FC Seeds

FC flour described in Section 2.1 (60 g) was soaked in 600 mL of buffer A, which contained 5 mM acetate-LiOH (pH 5.0, 4 °C) for 20 min under stilled conditions at 5 °C. After centrifugation at 20,300× *g* for 20 min, supernatant was applied to an anion exchange chromatography (“Q-sepharose Fast Flow”, Cytiva Tokyo, Japan) in which gel was equilibrated with buffer A and manually packed into a 20 × 100 mm syringe. After washing with 100 mL of buffer A, proteins were eluted using a linear 400 mL of gradient of 5 mM to 1 M LiCl in buffer A. Active fractions were collected and loaded onto a gel filtration chromatography column (“HiLoad 16/600 Superdex 200pg”, Cytiva Tokyo, Japan) equilibrated with buffer A. Active fractions were collected and diluted with 10 times volume distilled deionized water and then applied to an anion exchange chromatography again (“Mono Q 5/50 GL”, Cytiva Tokyo, Japan). After washing with 20 mL of buffer A, proteins were eluted using a linear 40 mL of gradient of 5 mM to 400 mM LiCl in buffer A. Active fractions were collected and diluted again with 10-fold the volume of distilled deionized water and then subjected to anion exchange chromatography (“Mono Q 5/50 GL”, Cytiva Tokyo, Japan). After washing with 20 mL of buffer A, proteins were eluted using a linier 40 mL gradient of 80 mM to 120 mM LiCl in buffer A. In each purification step, rutinosidase activity was measured under standard assay conditions. A standard assay was performed to measure the rutin and quercetin concentrations in the reaction mixture using UPLC. The standard assay mixture consisted of 50 mM acetate–LiOH buffer (pH 5.0, 4 °C), 20% (*v*/*v*) methanol, and 0.2% (*w*/*v*) rutin in a final volume of 0.05 mL. The reaction was performed at 25 °C and then stopped by the addition of 0.2 mL methanol. To conveniently detect f3 g activity, the fluorescence intensity of quercetin (the aglycone moiety of rutin or isoquercitrin) in a reaction mixture spotted on paper (Toyo No. 1) was detected by UV light (360 nm) (AlphaImage MINI, ATTO, Tokyo, Japan) [15]. The protein concentration of each purification step was measured using the improved method of Bradford [16] using bobbin serum albumin as a standard protein.

The active fractions were collected and subjected to enzymatic characterization. To investigate the optimal pH of the enzyme, rutinosidase activity using rutin as substrates was determined at different pH: 2.0 (200 mM glycine–HCl buffer), 3.0–4.0 (200 mM citrate–LiOH buffer), 5.0–6.0 (200 mM acetate–LiOH buffer), 7.0 (200 mM imidazole–LiOH buffer), 8.0 (200 mM tris–HCl buffer), or 9.0–10.0 (200 mM borate–LiOH buffer). To assess optimal temperature, the enzyme solution was pre-incubated for 30 min at temperatures ranging 5–75 °C. Thereafter, rutinosidase activity was measured under standard assay conditions, except for the reaction temperature (reaction was performed at the same temperature as pre-incubated). The *K*_m_ values were determined using Lineweaver–Burk plots at different substrate concentrations. The above measurements were performed in three independent replicates.

### 2.4. In-Gel Detection of Rutinosidase in Cultivated Buckwheat Varieties

In-gel detection was performed using a rutin–copper complex [17]. Buckwheat flour (10 mg fresh weight flour) was homogenized with 1 mL of buffer A using a motor. A crude enzyme solution was obtained through 20,300× *g* centrifugation and subjected to native-PAGE after addition of 10% (*v*/*v*) glycerol. Native-PAGE was performed using 0.75 mm thickness of 4.5% stacking and 7.5% separation polyacrylamide gel under non-denaturing conditions using a slab gel apparatus (“Mini protean Tetra cell”, Bio-Rad Laboratories, Heracles, CA, USA).

After electrophoresis under constant 100V condition, the gel was equilibrated with 50 mM acetate–LiOH buffer (pH 5.0, 4 °C) containing 20% (*v*/*v*) methanol for 10 min under horizontal shaking condition at 50 rpm. Then, the equilibrated gel was stained with 50 mM acetate–LiOH buffer (pH 5.0, 4 °C), 20% (*v*/*v*) methanol, 0.6% (*w*/*v*) rutin, and 5 mM CuSO_4_ for 60 min under horizontal shaking condition at 50 rpm. When the yellow-brown bands were visualized, the gel was removed from the staining buffer and washed with water for 60 min. To obtain clear staining images, we considered a sample volume corresponding to 5–20 mg of protein per lane.

### 2.5. Investigation of Rutin and Quercetin Concentration in FC Dough and Evaluation of Bitterness

One gram of FC flour obtained in Section 2.1 was placed in test tubes and then incubated at 25 °C for 3 h. Water incubated at the same temperature for the same duration was added to each tube (2 mL) to make doughs. After water addition, the flour and water were immediately mixed using a spatula for one minute to obtain uniform dough. The dough was then placed in a 25 °C incubator (HB-80, TAITEC, Saitama, Japan) for 10 min. Next, 9 mL of methanol containing a 20% volume of 0.1% (*v*/*v*) phosphoric acid was added to the dough, and samples were subjected to extraction of rutin and quercetin at 80 °C for 3 h. After extraction, the centrifuged supernatant was analyzed using UPLC [11], and the rutin and quercetin contents in the doughs were determined. To evaluate the bitterness of FC, sensory analysis was performed by six expert panelists, who consisted of men ranging 48–63 years old and women ranging from 30 to 50 years old. For the analysis, each panelist placed FC flour or FE flour (1.0 g) in their mouth and tasted the samples for 1 min. The panelists assessed the bitterness of each flour sample and classified the samples based on the degree of bitterness into two categories: bitter (bitter or extremely bitter) or not bitter (no or little bitterness). We also prepared steam-heated flour to inactivate rutinosidase completely. Approximately 10 g of FC flour was packed in a non-wooden bag for 1 cm and steam-heated for 30 min in a steamer. Steam-heated flour was also used for the above-mentioned dough-making test and sensory analysis.

## 3. Results

### 3.1. Organ Distribution of Rutin and Rutinosidase Activity

**Figure 1:**
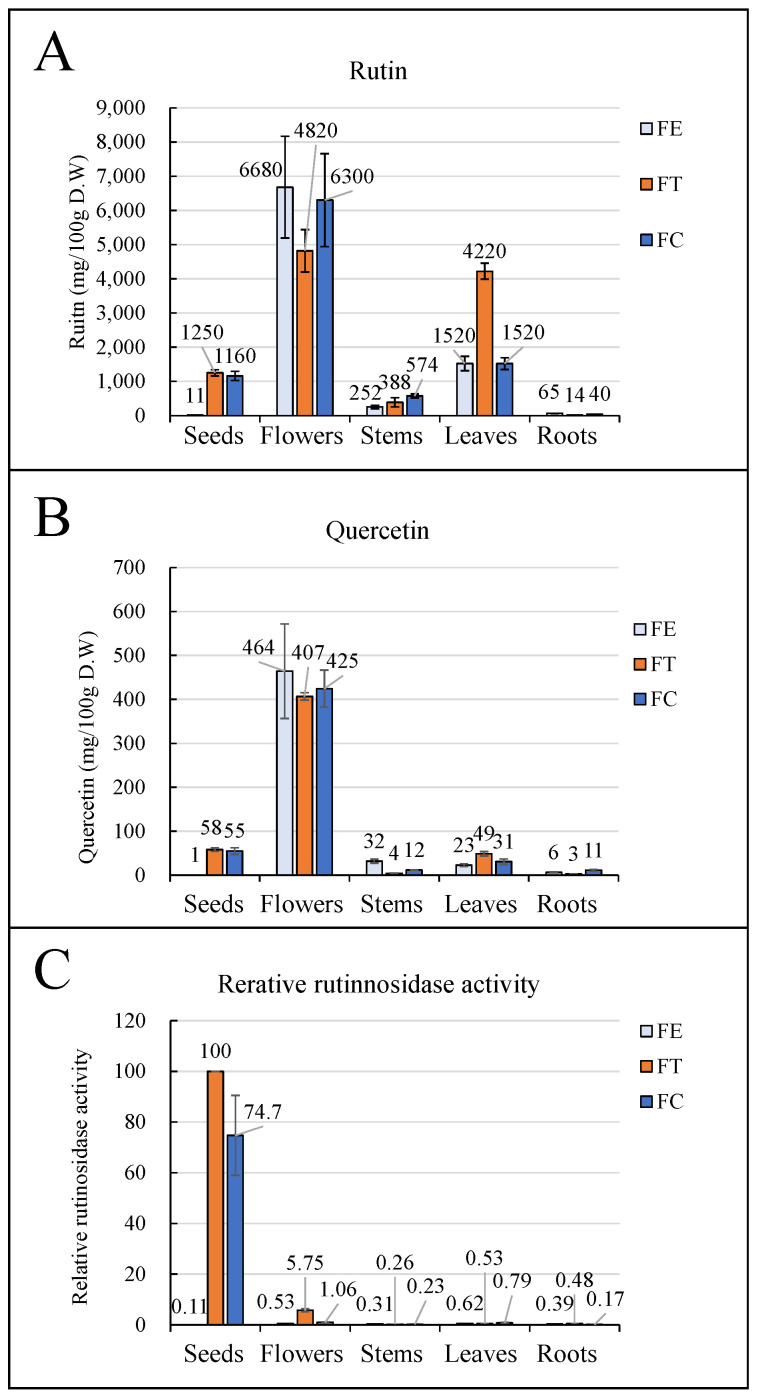
Rutin and quercetin concentration and Rutinosidase activity in different organs in FE, FC, and FT. Rutin (**A**) and quercetin (**B**) concentrations were evaluated using UPLC. To investigate the in vitro rutinosidase activity (**C**), a standard assay was performed by measuring the quercetin concentration in the reaction mixture using UPLC. Data represent the mean of independent experiments (*n* = 3). Bars indicate SD.

Common (FE), tartary (FT), and FC (FC) had the highest rutin concentrations in flowers compared to other organs (Figure 1A). Leaves had higher rutin concentrations in all buckwheat species, followed by flowers. Among them, FT leaves had the highest rutin concentration. Additionally, the concentrations of rutin in FE and FC flowers were in the range of 18,700–42,300 and 41,200 mg/100 gDW, respectively. In seeds, FC and FT accumulated approximately the same level of rutin. For FT, rutin-rich foods with high antioxidative activity have been developed [18]. Therefore, FC seeds would also be beneficial for these foods in terms of rutin-rich food material. In contrast, FE had one hundredth the level of rutin compared to them. This result is consistent with those of previous studies [11]. In all buckwheat species and organs, rutin was the major flavonoid in the extract. Quercetin concentrations were also higher in the flowers of all buckwheat varieties (Figure 1B). In contrast, rutinosidase activity was higher in FT seeds, whereas FE was almost zero (Figure 1C). This result is consistent with that of a previous study [11]. FC seeds also contain high rutinosidase activity, which is the first report of FC, suggesting that rutin hydrolysis may occur in FC flour. In FC seeds, most rutinosidase was distributed in the testa (Figure S2).

### 3.2. Purification and Characterization of Rutinosidase from FC Seeds

**Figure 2:**
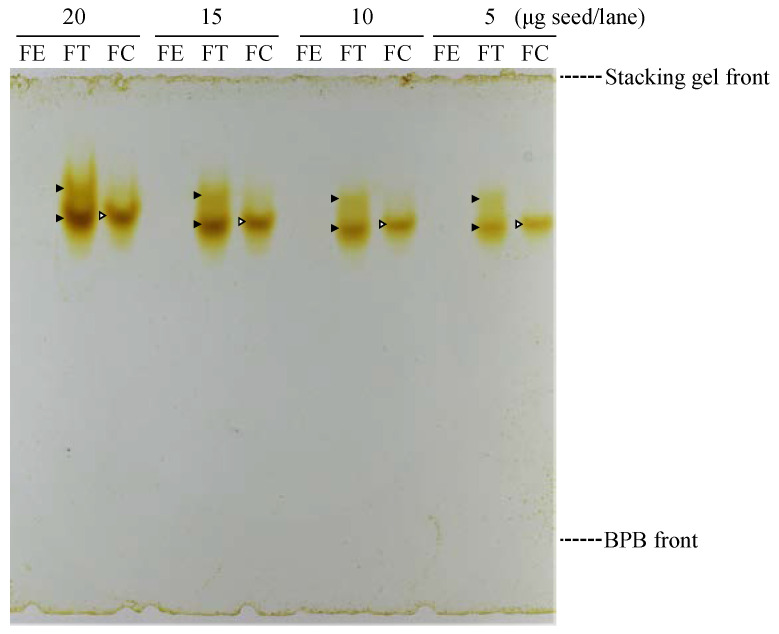
In-gel detection of rutinosidase isozyme in FE, FT, and FC seeds. In-gel detection was performed using a rutin–copper complex. To obtain clear staining images, we considered a sample volume corresponding to 5–20 μg seeds per lane.

**Figure 3:**
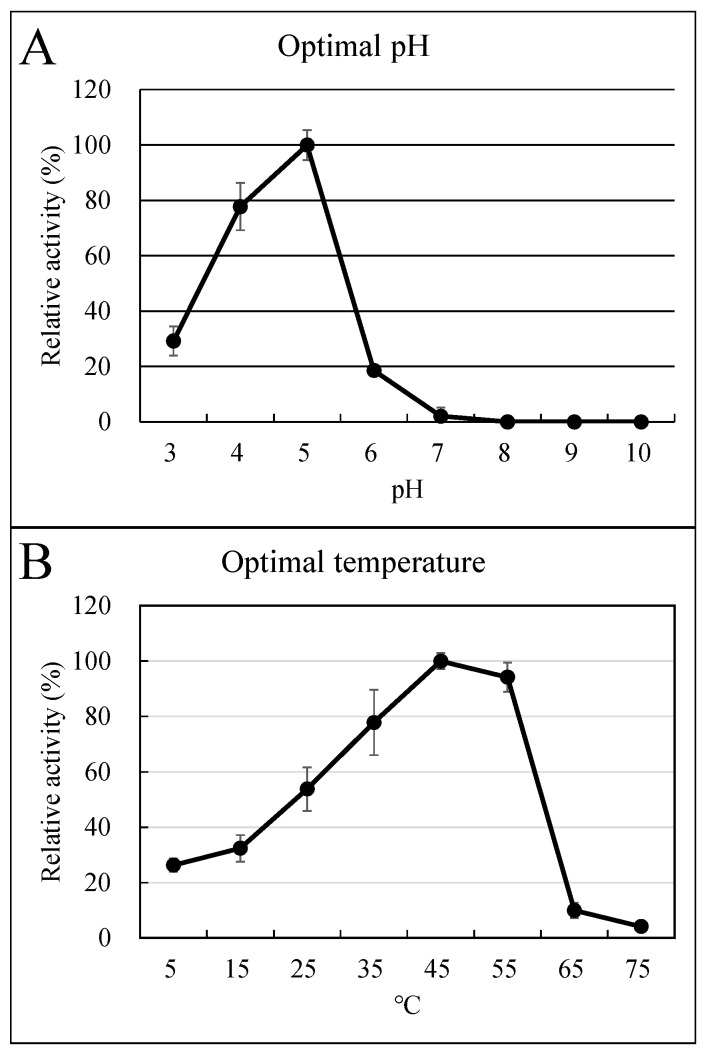
Optimal temperature and pH of purified FC rutinosidase. Optimal pH (**A**) of rutinosidase activity in homogenous purified using rutin as substrates was determined at different pHs: 2.0 (200 mM glycine—HCl buffer), 3.0 to 4.0 (200 mM citrate—LiOH buffer), 5.0 to 6.0 (200 mM acetate—LiOH buffer), 7.0 (200 mM imidazol—LiOH buffer), 8.0 (200 mM tris—HCl buffer), or 9.0 to 10.0 (200 mM borate—LiOH buffer). To assess optimal temperature (**B**), rutinosidase activity was measured at temperatures ranging 5–75° followed by 30 min pre-incubation of the enzyme solution. Data represent the mean of independent experiments (*n* = 3). Bars indicate SD.

We purified rutinosidase from FC seeds 46.1-fold likely to be homogeneous (Table 1, Figure S3). The final yield was 4.34%, and total protein was 0.19 mg. The purification fold was increased by ion exchange chromatography using Q-Sepharose and Mono Q step (5–400 mM) (Table 1). Although gel-filtration chromatography has a low purification fold, it is necessary to purify it to homogeneity. A combination of at least two chromatography methods based on different principles is effective for high purification. Mono Q anion exchange chromatography was used twice. This is because the second Mono Q step is performed under a low-range gradient (80–120 mM) to purify rutinosidase and to investigate the presence or absence of isoforms. The purified FC rutinosidase consisted of one isozyme in the in-gel detection method, whereas the FT rutinosidase consisted of two isozymes (Figure 2). The mobility of FC rutinosidase in Figure 2 was almost the same as that of the lower band of FT rutinosidase. The molecular weight of the FC rutinosidase was 58,400 ± 2700 based on three gel filtration measurements (Table 2). This result was similar to that of two isozymes (f3gI; 58,200) in FT rutinosidase [11]. FC rutinosidase activity was optimal at pH 5.0 and 45 °C (Figure 3A). In addition, rutinosidase activity was not detected over pH 8.0 (Figure 3A). This result resembles Tartary buckwheat rutinosidase (f3gI, f3gII; pH 5.0 and 40 °C). However, FC rutinosidase could retain a wider temperature range, such as under 5 and over 50 °C (Figure 3B). This information is useful for the application of FC rutinosidase in the synthesis of rutinosides and rutinose by reverse hydrolysis. The *K*_m_ for rutin in FC rutinosidase was 0.367 mM (Table 2), which was approximately three-fold higher than that of FT rutinosidase [11]. In addition, the Vmax value for rutin was 36.2 U/mg (Table 2), which is almost the same as that of FT rutinosidase. In contrast, rutinosidase in *Penicillium rugulosum* [19] consists of a homotetramer with a molecular weight of 245,000 and an optimal pH of 2.2. Therefore, the enzymatic characteristics of FC rutinosidase are highly similar to those of FT rutinosidase compared to rutinosidase in *P. rugulosum*.

### 3.3. Investigation of Rutin and Quercetin Concentration in FC Dough and Evaluation of Bitterness

Rutin content in dough rapidly decreased after the addition of water and became almost zero after 10 min (Table 3). Parallel to the decrease in rutin, quercetin increased (Table 3). The decrease in rutin moles and increase in quercetin moles were almost the same, suggesting that increased quercetin was generated by rutinosidase activity. In the bitterness evaluation of flour, no panelists felt bitterness in common buckwheat, whereas all panelists reported strong bitterness in the FC flour. In addition, no panelists felt bitterness in the dough made from steam-heated FC flour. The dough pH of FC flour was determined as follows: 10 g of FC flour was mixed with 200 mL of deuterium-depleted water (DDW) in a stirred state, and the pH was measured 1, 10, 30, and 60 min after the addition of water.

## 4. Discussion

### 4.1. Organ Distribution of Rutin and Rutinosidase Activity

In this study, rutin concentration was higher in the flowers and leaves of FE, FT, and FC plants. In particular, in flowers, the rutin concentration was over 4800 mg/g D.W., which is high compared to other rutin sources such as seeds and stems. This result is consistent with prior research, wherein the concentrations of rutin in FE and FT leaves were approximately 40,000 and 100,000 mg/100 gDW, respectively [20]. Similarly, the concentration of rutin in FC leaves was approximately 40,000 mg/100 gDW [21]. Therefore, flowers and leaves are promising sources of rutin. Some studies have demonstrated that flowers and leaves of buckwheat contain the photo-toxic substance fagopyrin [22]. Therefore, it is necessary to remove fagopyrin from these organs before food use. On the other hand, FT and FC seeds contained more than 1000 mg/g D.W. (Figure 1). This result is consistent with prior research, indicating that the concentrations of rutin in FT seeds ranged from 1110 to 1950 mg/100 gDW. [23]. Compared to leaves and flowers, seeds contain a low percentage of water; therefore, they have an advantage in terms of drying cost. In addition, flour is useful for food processing, such as in seed grains such as kasha, noodles, and bread. Therefore, the high rutin concentrations in FT and FC seeds provide useful information. Rutin-rich food from FT has been shown to have a clinical effect on body weight loss [24]. Therefore, the FC seed probably had the same effect. Recently, it was reported that heat treatment of FT seeds provides dehulled and gelatinized products with denatured rutinosidase in a normal-rutinosidase variety [25]. Using a circulating fluidized bed at 200 °C, the buckwheat was observed to produce a popcorn-like gelatinized product. The rutin content was retained without the formation of the bitter product quercetin, owing to the denaturation of rutinosidase in the heat-treated products. Nutritional analysis of pre- and post-treated products showed retention of macro-and micronutrients even after heat treatment. The popped FT could therefore be obtained by a simple treatment and may reveal new opportunities to utilize pre-gelatinized and rutin-rich properties by rutinosidase denaturation. Therefore, FC seeds can also be used above rutin-rich materials in popcorn-like food materials. Quercetin has also been reported as a functional compound. However, the quercetin concentrations in FE, FT, and FC were one-tenth lower than that of rutin. Quercetin is an aglycone of rutin synthesized by glucosyl- and rhamnosyl-transferase activity against quercetin. Therefore, the low accumulation of quercetin was due to its position as an intermediate substance for rutin synthesis. Rutinosidase activity was detected in all organs in the FE, FT, and FC groups. In roots, FC has a perennial trait; therefore, its root growth is vigorous compared to that of the annual species FC and FE. The rutin concentration in annual-year roots in FC was not as high as that in other organs (Figure 1A). Therefore, for the next step, rutin and quercetin concentrations in roots grown after the 2nd year should be evaluated.

This is the first report to demonstrate the presence of rutinosidase activity in FC. Among the tested organs, high rutinosidase activity was detected in the FT and FC seeds. However, the seed rutin content in FT does not change for at least six years [11]. This is because of differences in organ distribution in rutin and rutinosidase in FE seeds; most of the rutin was distributed in the embryo and rutinosidase was in the testa [11]. On the other hand, once water was added to FT flour in which the embryo and testa were crushed and mixed, rutinosidase activity hydrolyzed rutin within a few minutes. In addition, rutin hydrolysis triggers bitter taste generation in FT [16]. The FC seeds also contained high amounts of rutin in the embryos and testa (Figure S2A) and rutinosidase (Figure S2B). Therefore, we conclude that rutin hydrolysis and bitterness generation could also be caused by FC. On the other hand, certain secondary metabolites may be associated with bitterness. Therefore, future studies should investigate the bitterness potency of a dilute solution of quinine. In FT, the flour milling percentage is affected by the moisture content [26]. Briefly, the crushed grains were divided into flour, bran, and residue by sieving. The rutin contents of flour and bran were in the range of 360–2060 and 3032–8649 mg/100 g D.W., respectively. When the grain moisture content was low, the flour and bran milling percentages were high. The rutin content of the flours also increased with bran milling percentage. On the other hand, the rutin content of bran attained maximum values when grain moisture contents were in the range of 10–16%. This indicates that the rutin content of flour and bran can be controlled by adjusting the grain moisture content prior to roll milling. Therefore, it should be possible to control bran rutin concentration using the same method in FC.

### 4.2. Purification and Characterization of Rutinosidase from FC Seeds and Contribution of Rutinosidase on Rutin Hydrolysis and Bitterness in FC Dough

FC rutinosidase has a high affinity for rutin (Table 2), similar to that of FT rutinosidase. FC and FT seeds contained a large amount of rutin (Figure 1). The low *K*_m_ of these rutinosidases is suitable for hydrolyzing rutin in seeds. The FC rutinosidase intensity in vitro was high enough to hydrolyze rutin in its seed within several seconds under optimal reaction conditions. Therefore, we hypothesized that rutin in FC seeds would be hydrolyzed immediately after the addition of water. In an investigation of rutin hydrolysis in the dough of FC flour, rutin in FC flour was almost completely hydrolyzed 10 min after the addition of water (Table 3). Parallel to rutin hydrolysis, quercetin content increased (Table 3). From these results, we conclude that rutin hydrolysis in FC flour occurs via rutinosidase activity, which is also present in FC flour. In addition, the fact that all panelists experienced strong bitterness supports the idea that quercetin, which is generated by the rutinosidase activity of rutin, is an important cause of strong bitterness. Generally, 10 min is required to produce noodles from flours. Thus, 10 min was sufficient to hydrolyze rutin in the dough of FC flour. Rutin is one of the most important functional compounds in FC seeds. Therefore, to prevent rutin hydrolysis, it is important to take advantage of FC-containing food. In addition, preventing the hydrolysis of rutin is important for preventing the development of bitterness. Below, we discuss how to prevent rutin hydrolysis based on the enzymatic characteristics of FC rutinosidase. The pH of FC flour was approximately 6.2 (Figure S4). On the other hand, the optimal pH of FC rutinosidase was approximately 5.0, whereas rutinosidase activity was weak (>8.0). Therefore, the control of dough pH may be useful in preventing rutinosidase activity in dough. To date, some pH-adjustable compounds have been developed, such as sodium bicarbonate. In FT dough, rutinosidase activity is too strong to prevent rutin hydrolysis by the addition of sodium bicarbonate [27]. On the other hand, in the trace-rutinosidase variety developed by our research group [13], most parts of rutin hydrolysis were prevented through the addition of sodium bicarbonate; addition of 0.125% sodium bicarbonate meant that the rutin content for all doughs remained >80% at 60 min after addition of water. This would provide sufficient time to make and cook noodles in a home or factory environment. However, handmade noodle makers sometimes store raw noodles for more than 480 min. In addition, bread making requires a fermentation procedure that can take several hours. In such cases, addition of a 0.125–0.5% solution of NaHCO_3_ should be effective. In bread dough (100% water to flour ratio), the rutin residual ratio is also affected by the blending ratio of FT flour to wheat flour; a low blending ratio tends to result in a high rutin residual ratio in noodles. In galette dough (containing a 400% water to flour ratio), only the 1.0% NaHCO_3_ solution retained a rutin residual ratio of 80% for >120 min after addition of water. In the trace-rutinosidase variety in FT, rutin concentration in foods was highly retained without heating or addition of NaHCO_3_. Therefore, to prevent rutin hydrolysis in FC, interspecific hybridization between FC and the trace-rutinosidase variety of FT is also effective. Recently, breakthroughs in the interspecific cross-breeding of buckwheat have been made [28,29]. These reports support this theory.

In contrast, roasting can prevent rutin hydrolysis in FT bran [30]. Although this roasting treatment causes denaturation of protein and starch, it is effective in eliminating rutinosidase activity by using bran as a rutin-rich material for tea beverages, similar to rutin-rich food additives. Rutin content demonstrated, during successive infusions of roasted FT bran and grain, to develop functional FT tea. Samples (6 g) of roasted FT bran and grain were rinsed with 300 mL of hot water (>95 °C) for 0.5 min. For the first infusion test, the tea infusion sample of roasted FT bran contained a distinctly higher amount of rutin (389 mg/L) than the roasted FT grain (68 mg/L). Overall, rutin was more effectively extracted from roasted FT bran than roasted FT grains. Therefore, roasted FC bran could also be used in the same manner as FT.

## 5. Conclusions

FC seeds and leaves are promising sources of rutin. FC rutinosidase has a low Km for rutin, and its optimal pH is the same as that of dough. Once water was added to the flour, rutin was hydrolyzed within 10 min, by which quercetin was generated parallel to rutin decomposition. In addition to rutin hydrolysis, strong bitterness is generated. These results indicate that FC rutinosidase causes rutin hydrolysis and triggers bitterness in FC dough. The most important quality issues in the industrial use of FC are rutin hydrolysis and the generation of strong bitterness. In this paper, we propose that foods with high rutin content and no bitterness can be produced by manufacturing them under the conditions in which rutinosidase is not active.

## Figures and Tables

**Table 1:** Summary of purification of rutinosidase from FC seeds.

	Volume	Total Protein	Total Activity	Specific Activity	Purification	Yield
Purification step	(mL)	(mg)	(μkat)	(μkat/mg protein)	(fold)	(%)
Crude extract	542	196	553	2.82	1.00	100
Q-Sepharose Fast Flow	24.0	26.9	295	11.0	3.89	53.3
Hiload16/600 Superdex 200pg	18.0	5.18	115	22.3	7.90	20.9
Mono Q 5/50 GL (5–400 mM LiCl)	7.0	0.33	28.5	86.4	30.6	5.15
Mono Q 5/50 GL (80–120 mM LiCl)	7.0	0.19	24.0	130	46.1	4.34

**Table 2:** Kinetic constants of purified rutinosidase for rutin.

*K*_m_	(mM)	0.367 ± 0.0235
Vmax	(U/mg)	36.2 ± 1.72
Data are means ± SD (*n* = 3).		

**Table 3:** Relative rutin and quercetin concentration in FC dough.

	Minutes after Addition of Water to Flour	
	0	10
Rutin	100 ± 2.40	0.160 ± 0.029
Quercetin	0.014 ± 0.004	100 ± 8.03
Data are means ± SD (*n* = 3) Dough was stored at 25 °C Rutin at 0 min = 100 Quercetin at 10 min = 100		

## Data Availability

Data will be made available on request.

## Supplementary Materials

The following supporting information can be downloaded at: https://www.mdpi.com/article/10.3390/foods12071417/s1, Figure S1: Picture of FC and flow chart of experiments; Figure S2: Tissue distribution of rutin and rutinosidase activity in FC seed; Figure S3: Chromatogram of final purification step in rutinosidase purification; Figure S4: Changes of pH in FC dough.
