# Supplementary material for: Rutin Concentration and Characterization of Rutinosidase in Perennial Buckwheat (Fagopyrum cymosum) and Its Application in Foods
Source: Foods. 2023 Mar 27;12(7):1417. doi: 10.3390/foods12071417 (PMC10093837; doi:10.3390/foods12071417)
Supplement: Supplementary file 1 [file foods-12-01417-s001.zip › foods-2301832-supplementary.pdf]

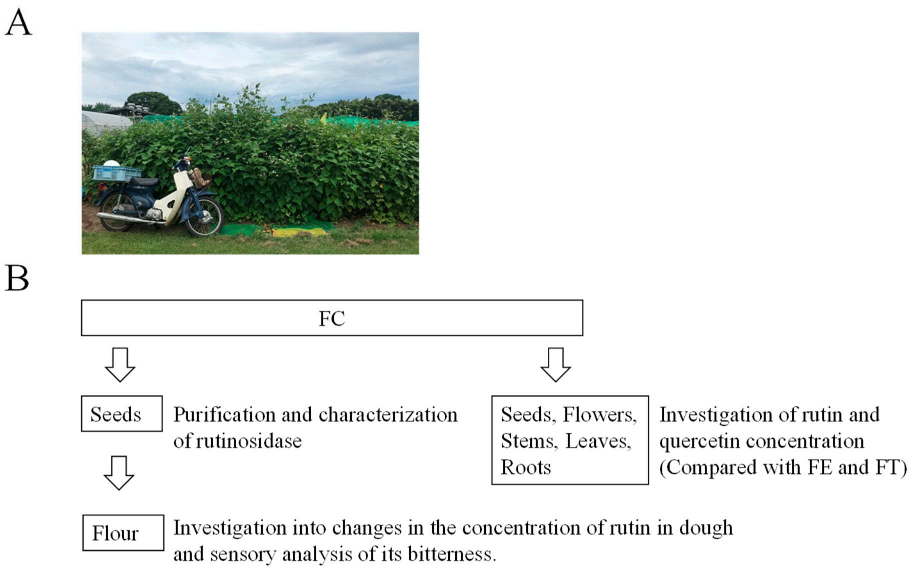


**Fig. S1.** Picture of FC and flow chart of experiments.

A; picture of FC plant, B; flow chart of experiments

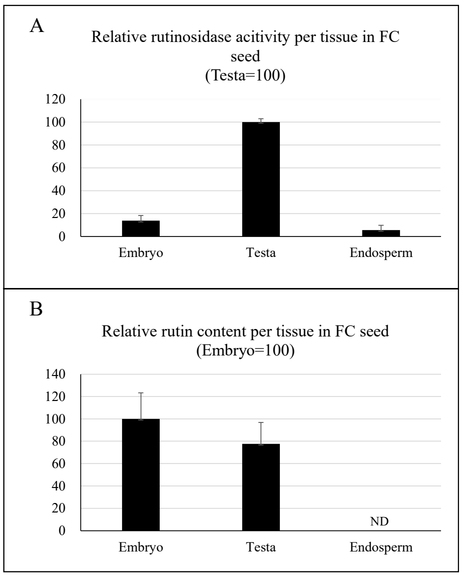


**Fig. S2.** Tissue distribution of rutin and rutinosidase activity in FC seed. To investigate the tissue distribution of FC rutinosidase in seeds, testa, endosperm, and embryo were separated using tweezers and applied to the analysis of rutin concentration and rutinosidase activity. Data represent the mean of independent experiments (n=3). Bars indicate SD.

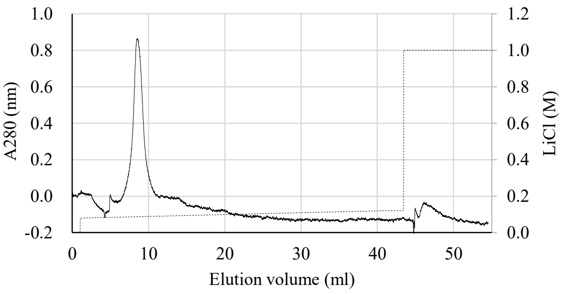


**Fig. S3.** Chromatogram of final purification step in rutinosidase purification. The final purification step was performed using anion exchange chromatogram under an 80-120-mM gradient using LiCl (right scale). Protein content was monitored at A280 (left scale).

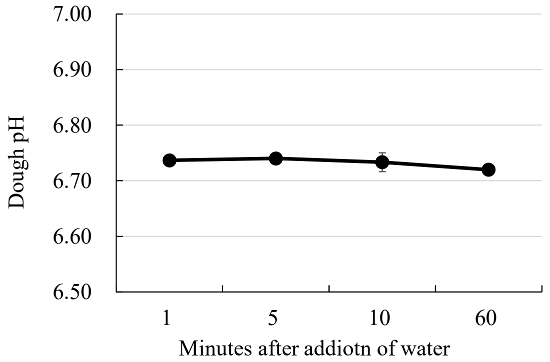


**Fig. S4.** Changes of pH in FC dough. To evaluate changes in dough pH in FC flour, 10 g of FC flour was mixed with 200 mL of DDW in a stirred state, and the pH was measured at 1, 10, 30, and 60 min after the addition of water. Data represent the mean of independent experiments (n=3). Bars indicate SD.
